# Effectiveness of the surgical torque limiter: a model comparing drill- and hand-based screw insertion into locking plates

**DOI:** 10.1186/s13018-016-0458-y

**Published:** 2016-10-17

**Authors:** Christopher Ioannou, Matthew Knight, Luca Daniele, Lee Flueckiger, Ezekiel S. L. Tan

**Affiliations:** 1 Orthopaedic Department, Gold Coast University Hospital, 1 Hospital Blvd, Southport, QLD 4215 Australia; 2 Mechanical Engineering, Griffith University, Gold Coast, Queensland Australia

**Keywords:** Locking, Plate, Torque, Limiter, Screw, Drill, Hand

## Abstract

**Background:**

The objective of this study is to analyse the effectiveness of the surgical torque limiter during operative use. The study also investigates the potential differences in torque between hand and drill-based screw insertion into locking plates using a standardised torque limiter.

**Methods:**

Torque for both hand and power screw insertion was measured through a load cell, registering 6.66 points per second. This was performed in a controlled environment using synthetic bone, a locking plate and locking screws to simulate plate fixation. Screws were inserted by hand and by drill with torque values measured.

**Results:**

The surgical torque limiter (1.5 Nm) was effective as the highest recorded reading in the study was 1.409 Nm. Comparatively, there is a statistically significant difference between screw insertion methods. Torque produced for manually driven screw insertion into locking plates was 1.289 Nm (95 % CI 1.269–1.308) with drill-powered screw insertion at 0.740 Nm (95 % CI 0.723–0.757).

**Conclusions:**

The surgical torque limiter proved to be effective as per product specifications. Screws inserted under power produce significantly less torque when compared to manual insertion by hand. This is likely related to the mechanism of the torque limiter when being used at higher speeds for which it was designed. We conclude that screws may be inserted using power to the plate with the addition of a torque limiter. It is recommended that all screws inserted by drill be hand tightened to achieve adequate torque values.

## Background

Locking plates are widely used in orthopaedic surgery and are highly effective in the osteosynthesis of fractures [1, 2]. The use of drill power to insert and lock screws into these plates is often advised against by manufacturing guidelines [1, 2] and is often reiterated anecdotally through both company representatives and surgeons alike. The risks are believed to be related to over tightening through excessive drill power and include screw head stripping, screw breaking, potential cold welding and subsequent difficult removal [3–5].

The increased time required for insertion of long screws by hand into locking plates raises questions around surgical efficiency and surgeon fatigue. Despite potential benefits through efficiency of drill-based screw insertion, there appears to be little evidence evaluating potential differences in applied torque between the two modes of insertion and on their relative impact on construct strength and risk of complications.

A literature review found no studies comparing insertional torque differences between hand and drill power when using a standardised torque limiter, and a number of implant manufacturers could not supply this data to us readily. Our aim was to quantify and compare peak insertion torque values using a 1.5-Nm surgical torque limiter in an attempt to further define optimum method of screw insertion and better consider risk to a locking plate construct. Our hypothesis was that a locking screw inserted under drill power will generate higher insertional torque and consequently put the locking screw interface at risk of over tightening.

## Methods

**Fig. 1:**
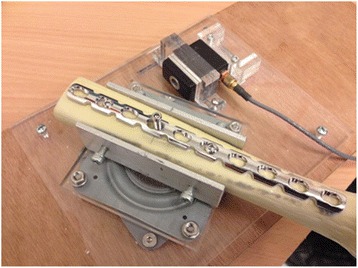
LCP reconstruction plate fixed onto Synbone on a rotating platform adjacent to the load cell

In collaboration with the Griffith University Mechanical Engineering Laboratory, a construct was created to replicate the surgical process of screw and plate fixation to bone. A synthetic model (*Synbone*) was used to simulate bone with a Synthes LCP Reconstruction Plate fixed with two screws equidistant from the central screw. The model was secured onto a rotating platform (Fig. 1). Rotation of the platform resulting from screw insertion torque was transmitted to a load cell which was calibrated using a known weight of 50 g. The cell had a maximum of 2 kN and produced an output in volts. A digital multimeter then logged results to the computer with the use of the Labview program recording 2000 points for a time period of 300 s. This equated to a sample rate of 6.66 points per second. With a known fixed lever arm, voltage (V) was converted into torque (Nm).

Screw insertions were performed through the same drill hole at a predetermined distance from the load cell. Testing was performed in a controlled environment with all procedures undertaken by a single surgeon.

The first round of testing analysed the insertion of the locking screw to the plate by hand. A 3.5 mm × 20 mm locking screw was inserted with a standard small fragment screw driver (Synthes) with torque limiter attachment. A standardised number of five audible clicks, representing triggering of the torque limiter five times, were used for each screw insertion to ensure appropriate capture of the insertional torque forces generated. This process was repeated ten times to increase statistical power.

The second round of testing analysed screw insertion under power, also with 50 clicks recorded from ten separate screw insertions. A trauma drill (Stryker, CD3) was used with the torque limiter attached. The maximum RPM for the drill was measured using a tachometer at 1506 RPM. Care was taken to create a controlled speed of insertion of the screw; however, technically, this was more difficult. Audible clicks were again used as in surgical practice.

## Results

From the data recorded, peak insertion torque values were calculated for the different groups. Analysis of the data demonstrated that the surgical torque limiter, rated at 1.5 Nm, was effective in our simulated operative setting. The highest recorded torque value was by hand at 1.409 Nm. Comparatively, the highest recorded value at insertion under power was 0.898 Nm.

**Fig. 2:**
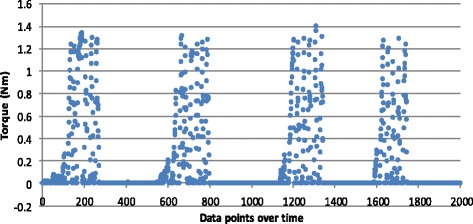
Torque measurements for a single screw inserted into a plate by hand four times. Each screw insertion demonstrating five triggers (audible clicks) of the torque mechanism

**Fig. 3:**
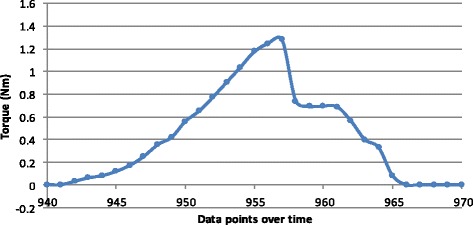
Focused torque values for a single trigger of the torque mechanism (click) of a screw inserted by hand

For the screws inserted by hand, data points clustered in peaks for each separate screw insertion. As seen in Fig. 2, four separate screw insertions are shown with each peak representing the torque recorded for five audible clicks. Closer observation of each separate triggering of the torque mechanism within a cluster of data for one screw insertion shows a clear rise and fall in torque values (Fig. 3). Individual screw insertion achieving five audible clicks was repeated ten times. The mean torque value for hand insertion (*n* = 50) was 1.289 Nm with a standard deviation (SD) of 0.069 (95 % confidence interval (CI) 1.269–1.308).

**Fig. 4:**
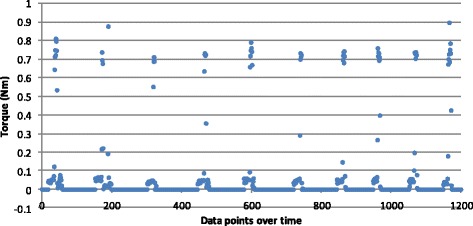
Torque measurements for a single screw inserted into a plate by power ten times. Each screw insertion demonstrating five triggers (audible clicks) of the torque mechanism

Torque values generated by screw insertion under power also relied on five audible clicks to capture the force produced for each screw insertion. As seen in Fig. 4, the data points for drill-powered insertion showed closer clustering for each screw insertion into the plate. Also shown is a lower maximum torque. The mean torque value for power insertion (*n* = 50) was significantly less at 0.740 Nm with a SD of 0.045 (95 % CI 0.723–0.757). The difference between the two groups was statistically significant (*p* = 0.0001).

**Fig. 5:**
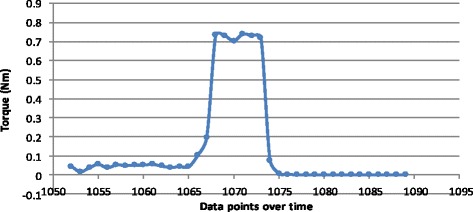
Focused torque values for a single screw inserted by power, demonstrating triggering of the torque mechanism five times

Closer focus on the torque patterns of individual screw insertion showed that the maximum torque in the power group (Fig. 5) was achieved more rapidly than in the hand group. As seen in Fig. 5, the maximum torque was reached within three data points (≈0.45 s) in contrast to Fig. 3, which took 16 points to achieve the same (≈2.4 s). Also observed for the torque values generated by hand insertion, after achieving maximum torque, the force quickly reduced to a plateau consistent with the average maximum (0.7 Nm) generated in the power group.

Throughout testing, we did not observe any evidence of head or thread stripping, evident wearing, cold welding, screw breakage or difficulty with screw removal.

## Discussion

We acknowledge that our single user model was simplistic and this data is applicable only to the small fragment system torque limiter rated at 1.5 Nm (Synthes). Our study used a 3.5-mm-diameter screw with a locking plate system and a synthetic bone model to simulate surgical fixation. Results may vary for different sized screws and constructs.

Our results proved the effectiveness of the torque limiter as no value exceeded the manufacturer specified limit of 1.5 Nm in any group. The study also demonstrated that the mean torque applied to a manually inserted locking screw (mean 1.2 Nm) is significantly greater (*p* < 0.001) than a screw inserted by drill (mean 0.7 Nm). This disproves our initial hypothesis and current belief that screws inserted by drill generate higher torque values and are at increased risk of over tightening or damage.

The higher maximum torque values generated in the hand group appear to be related to the gradient at which the screw approaches the torque limit. The increased speed in the drill (measured at a maximum of 1506 RPM) likely facilitates an early triggering of the torque limiting mechanism, limiting the torque to a subpar 0.7 Nm rather than the specified maximum 1.5 Nm. This raises concerns over the longevity and effectiveness of the torque limiter when used at higher speeds for which they may not have been designed.

Our results have shown significantly less insertional torque values when using the drill. This would suggest a lowered risk from increased torque and theorised damage to the involved construct. Supposed benefits could be reduced operating time and surgeon fatigue by inserting locking screws to a plate under power rather than hand.

While locking plates do not require the same levels of torque to be generated as their non-locking counterparts [2], they are still at risk of screw loosening with lower torque values [6]. This suggests that compared to hand-inserted screws, those inserted by drill are potentially at an increased risk of screw loosening and ultimate failure. This was not the focus of the study however and we did not evaluate the mechanical strength or properties of the final construct.

Further testing using a cadaveric model may be useful in better replicating a true model. It is also not known what impact large fragment systems, variable angle technology, different drills, plate interfaces, and varying screw size or length would have on torque values and could potentially be the interest of further study. Due to differences in torque limiter internal mechanism design between manufacturers and instrument sets, these results are not generally applicable to all torque limiting devices and other devices may behave quite differently. We advise caution when extrapolating these results to equipment different to those considered in this study.

Based on these findings, we suggest that locking screws can be inserted to the plate under power with a torque limiting attachment. As the insertional torque values produced are significantly less, it is therefore recommended that final screw tightening be performed by hand. The recommendation by companies not to use power for locking of screws may be based on risks of under-tightening or internal mechanism wear rather than over-tightening.

## Conclusions

In our model, we found that the surgical torque limiter is effective at preventing excess torque as per specifications. As less torque is generated by drill-powered insertion, we advise that screws can be powered directly into a plate without the risk of excessive torque. It is highly recommended that all screws inserted by drill be hand checked to ensure adequate torque and to decrease the risk of construct failure.

## Abbreviations

CI: Confidence interval
LCP: Locking compression plate
SD: Standard deviation
